# Facteurs de risque de la tuberculose multi-résistante dans la ville de Kinshasa en République Démocratique du Congo

**DOI:** 10.11604/pamj.2016.23.157.6137

**Published:** 2016-04-06

**Authors:** André Misombo-Kalabela, Georges Nguefack-Tsague, Ginette Claude Mireille Kalla, Emmanuel Afane Ze, Kimpanga Diangs, Tshapenda Panda, Ilunga Kebela, Serge Bisuta Fueza, Nzanzu Magazani, François-Xavier Mbopi-Kéou

**Affiliations:** 1 Ministère de la Santé Publique, République Démocratique du Congo; 2 Université de Yaoundé I, Faculté de Médecine et des Sciences Biomédicales, Yaoundé, Cameroun; 3 Université de Kinshasa, Faculté de Médecine, Département de Santé Publique, République Démocratique du Congo

**Keywords:** Tuberculose multi résistante, facteurs de risque, Kinshasa, République Démocratique du Congo, Multidrug-resistant tuberculosis, risk factors, Kinshasa, Democratic Republic of Congo

## Abstract

**Introduction:**

L'objectif de cette étude était de déterminer les facteurs de risque associés à la tuberculose multi résistance à Kinshasa en République Démocratique du Congo.

**Méthodes:**

Il s'agissait d'une étude cas témoins. Les cas comprenaient tous les patients tuberculeux résistants à la rifampicine et à l'isoniazide notifiés à Kinshasa de janvier 2012 à juin 2013. Les témoins étaient les patients tuberculeux traités durant la même période que les cas et qui à la fin du traitement étaient déclarés guéris. Pour cette étude, nous avons obtenu une clairance éthique.

**Résultats:**

L’échantillon était constitué de 213 participants dont 132 hommes (62%) et 81 femmes (38%). L’âge médian était de 31ans (16-73 ans). Les facteurs associés significatifs (p< 0,05) à la tuberculose multi résistante étaient le non-respect des heures de prise de médicaments (0R = 111) (80% chez les cas et 4% chez les témoins), l’échec au traitement (0R = 20) (76% chez les cas et 13% chez les témoins); la notion de tuberculose multi résistante dans la famille (0R = 6.4) (28% chez les cas et 6% chez les témoins); la méconnaissance de la tuberculose multi résistante (0R = 3.2) (31% chez les cas et 59% chez les témoins); un séjour en prison (0R = 7.6) (10% chez les cas et 1% chez les témoins) et l'interruption du traitement (0R = 6.1) ( 59% chez les cas et 19% chez les témoins).

**Conclusion:**

L’émergence de la tuberculose multi résistante peut être évitée par la mise en place des stratégies de diagnostic et de traitement appropriées.

## Abstract (en)

**Background:**

The aim of this study was to determine the risk factors for multidrug-resistant tuberculosis (TB) in the city of Kinshasa in the Democratic Republic of Congo.

**Methods:**

This was a case control study. The cases included all TB patients notified as resistant to rifampicin and isoniazid in Kinshasa from January 2012 to June 2013. The controls included TB patients treated during the same period as the cases and declared cured at the end of treatment. For this study, we obtained ethical clearance.

**Results:**

The sample consisted of 213 participants, 132 men (62%) and 81 women (38%). The median age was 31 years (16-73 years). Factors associated with significant (p< 0,05) multidrug-resistant tuberculosis were the non-observance of the hours of taking drugs (0R = 111) (80% cases, 4% controls), the failure of treatment (0R = 20 (76% cases, 13% controls); the concept of multidrug-resistant tuberculosis in the family (0R = 6.4) (28% cases, 6% controls); a lack of knowledge of multidrug-resistant tuberculosis (0R = 3.2) (31% cases, 59% controls); a stay in prison (0R = 7.6) (10% cases, 1% controls) and the interruption of treatment (0R = 6.1) (59% cases, 19% controls).

**Conclusion:**

The emergence of multidrug-resistant tuberculosis can be avoided by the installation of suitable diagnosis and treatment strategies.

## Introduction

La Tuberculose est une maladie infectieuse causée par *Mycobacterium tuberculosis* ou Bacille de Koch. Sa transmission est toujours directe, du sujet malade bacillifère au sujet réceptif, par voie aérienne du fait des bacilles contenus dans l'air, dans les gouttelettes de salive en suspension émises par le patient [1]. L'atteinte pulmonaire est la plus fréquente des localisations et représente la source habituelle de transmission La tuberculose représente un problème majeur de santé publique. Chaque année on compte environ 9 millions de nouveaux cas de tuberculose et près de 2 millions de personnes meurent de cette maladie [2]. Chaque année, près de 440 000 personnes contractent une tuberculose multi résistante et 150 000 personnes décèdent de cette forme de la maladie [3]. Son traitement est difficile et coûteux en raison de de la mauvaise réponse au traitement classique avec des médicaments de première intention. Les taux de guérison de la tuberculose multi résistante sont faibles (compris entre 50% et 70%) [2]. L'Afrique, avec ses 11% de la population mondiale, supporte à elle seule 27% du poids mondial de la tuberculose [2]. L'incidence de la tuberculose augmente chaque année de 6% et l’épidémie du VIH est la principale cause de cette augmentation. En effet, environ 30 à 50% des tuberculeux en Afrique sont co-infectés par le VIH [4]. La République Démocratique du Congo (RDC), l'un des 22 pays les plus atteints au monde, occupe le 5è rang en Afrique et le 11^ème^ rang dans le monde [2]. L'incidence en RDC de la tuberculose pulmonaire à microscopie positive est estimée à plus de 160 cas pour 100.000 habitants [2]. La RDC figure aussi parmi les pays qui ont le plus grand nombre de malades co-infectés par la tuberculose et le VIH/Sida [5]. Une enquête sur la tuberculose multi-résistante menée à Kinshasa/RDC de 1998-1999 a montré une prévalence de 2.2% parmi les nouveaux cas de tuberculose pulmonaire à Microscopie positive [6]. Parmi les hypothèses émises dans cette étude, le non-respect des directives par les prestataires de soins et la mauvaise adhérence au traitement par les malades tuberculeux seraient responsables de la tuberculose multi résistante dans la ville de Kinshasa; mais ces pistes de réflexion ne sont encore que des hypothèses à confirmer. Par ailleurs, aucune étude n'a jamais été menée sur l'ensemble de la ville de Kinshasa, pour déterminer les facteurs de risque qui contribuent à l’émergence de la tuberculeuse multi résistance. Aussi, l'objectif de cette étude était de déterminer les facteurs de risque qui contribuent à l’émergence de la tuberculose multi-résistante à Kinshasa en République Démocratique du Congo.

## Méthodes

**Figure 1:**
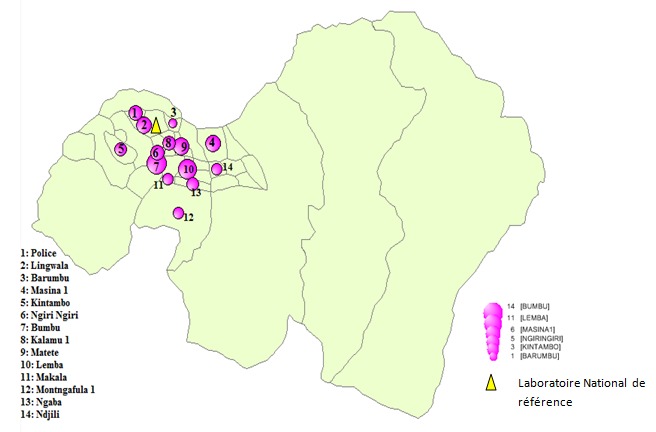
Distribution géographique de cas de tuberculose multi-résistante à Kinshasa en République Démocratique du Congo

Il s'agissait d'une étude analytique de type cas témoins réalisée de septembre à novembre 2013 auprès des patients enregistrés pour traitement pendant la période allant du janvier 2012 à Juin 2013 dans la ville de Kinshasa (Figure 1). Les cas étaient des patients atteints de tuberculose multi-résistante confirmés. Les témoins étaient les tuberculeux guéris qui avaient consultés au même moment et dans la même structure sanitaire que les cas. L’échantillonnage était exhaustif pour les cas et aléatoire simple pour les témoins (1 cas pour 2 témoins). Tous cas de tuberculose multi-résistante, absents de Kinshasa pendant l'enquête et ayant refusé de participer à l'enquête étaient exclus de l’étude. Nous avons obtenu le consentement éclairé de tous les participants à l′étude. Ces derniers étaient âgés de plus de 16 ans. Nous avons donc inclus 213 sujets (71 cas et 142 témoins) qui répondaient aux critères de l’étude. Les données collectées ont été saisies et analysées par des méthodes de statistiques descriptives et analytiques en utilisant le logiciel EPI INFO 7. Les différences entre les proportions ont été analysées en utilisant des tables de contingence et en appliquant le test de Chi-2. L′ampleur de l′association entre deux variables qualitatives a été évaluée par le rapport de côte pour déterminer les facteurs de risque pris individuellement (analyse uni variée). Les valeurs p < 0,05 étaient considérées comme statistiquement significatives.

## Résultats

***Caractéristiques sociodémographiques de la population d’étude:*** Le Tableau 1 représente les principales caractéristiques sociodémographiques de notre échantillon. La population étudiée était constituée de 132 personnes de sexe masculin (61,97%) et 81 personnes de sexe féminin (38,03%). Le sexe ratio femme/homme était 0,61. La moyenne d’âge des participants était de 33 ± 11ans avec un minimum de 16 ans et un maximum de 73 ans; le mode était de 28 ans et une médiane de 31ans. La population étudiée était en majorité sans profession 37,6% (80/213), 27,7% (59/213) avaient une profession autre que celle retenue dans le tableau (étudiant, ménagère, artiste musicien, soudeur, vendeur dans le restaurant appelé Malewa et vendeur dans une cabine téléphonique); 15% (32/213) étaient composés de fonctionnaires; la fonction libérale représentait 9% (20/213), 4,7% (10/213) étaient des commerçants et les chauffeurs représentaient 5,6% (12/213). Environ 51,6% des ménages (110/213) avaient une taille de moins de 6 personnes; 34.7% (74/213) de 7 à 10 personnes et 13.6% (29/213) de plus de 10 personnes. La taille moyenne des ménages était de 2±0.7 personnes avec un minimum d'une personne et un maximum de 3 personnes (Tableau 2). Sur l'ensemble des personnes enquêtées 60,1% (128/213) n'avaient pas une source de revenus et 39,9% (85/213) avaient une source de revenus.

**Tableau 1:** Évolution des cas de tuberculose multi-résistante

Année	Nombre de nouveau cas TPM +	Nombre des suspect MDR	Nombre de MDR confirmé à la culture
2011	8.908	149	**60**
2012	9.119	84	**76**
2013	4.497	51	**14**
Total	**22.524**	**281**	**150**

MDR : tuberculose multi résistante ; TPM+ : tuberculose pulmonaire à Microscopie positive

**Tableau 2:** Caractéristiques sociodémographiques de la population étudiée

	Cas N = 71(%)	Témoin N = 142(%)	Total
**Sexe**			
Féminin	26 (37)	55 (39)	81(38,03)
Masculin	45 (63)	87 (61)	132(61,97)
**Tranche d’âge (ans)**			
16 à 36	43 (61)	93 (65)	136(63,8)
37 à 73	28 (39)	49 (35)	77(36,2)
**Profession**			
Chauffeur	5 (0,1)	7 (5)	12(5,6)
Commerçant	3 (4,2)	7 (5)	10(4,7)
Fonction Libérale	8 (11,2)	12 (8)	20(9,4)
Fonctionnaire	9 (13)	23 (16)	32(15,0)
Autre	21 (30)	38 (27)	59(27,7)
Sans profession	25 (35,2)	55 (39)	80(37,6)
**Taille Ménage**			
Moins de 6	34 (48)	76 (54)	110(51,6)
7 à 10	21 (30)	53 (37)	74(34,7)
Plus de 10	16 (23)	13 (9)	29(13,6)
**Etat Matrimonial**			
Célibataire	44(62)	82 (58)	126(59,2)
Divorce	7(10)	3 (2)	10(4,7)
Marie	17(24)	50 (35)	67(31,5)
Union Libre	1(1,4)	5 (4)	6(2,8)
Veuf	2(3)	2(1,4)	4(1,9)
**Niveau d’étude**			
Aucun	0 (0)	1 (0,7)	1(0,5)
Humanitaire	31(43)	51 (36)	82(38,5)
Primaire	5(7)	4(3)	9(4,2)
Secondaire	11 (15)	45(32)	56(26,3)
Universitaire	24 (34)	41(29)	65(30,5)
**Source de revenus**			
Oui	26(37)	59(42)	85(39,9)
Non	45(63)	83(58)	128(60,1)
**Nombre de repas par jour**			
1	20 (28)	25(18)	45(21,1)
2	38 (54)	90(63)	128(60,1)
3	13 (18)	27(19)	40(18,8)

***Profil clinique de la population étudiée:*** Le Tableau 3 et le Tableau 4 représentent le profil clinique de notre échantillon. Environ 93,9% de personnes enquêtées (200/213) avaient déjà souffert de tuberculose au moins une fois dans leur vie. Chez les cas 98.5% (70/71) avaient déjà souffert de la tuberculose tandis que chez les contrôles 61,0% avaient un antécédent de tuberculose. 85,4% (182/213) n'avaient pas la notion de tabagisme dans leur passé et 14.6% (31/213) étaient de fumeur. Dans notre population d’étude, 67.1% (143/213) ne buvaient pas d'alcool et 32,9% (70/213) avaient une notion de prise d'alcool dans leur passé. Sur l'ensemble des enquêtés, 84,5% (180/213) avaient de connaissance sur la tuberculose et 15,5% (33/213) ne connaissaient pas la définition de la tuberculose Nous avons trouvé que 57,7% (123/213) des enquêtées avaient une notion de la tuberculose dans leur famille et 41.3% (88/213) n'avaient pas de notion de la tuberculose dans la famille. Chez les cas 71.8% (51/71) avaient une notion de tuberculose dans la famille tandis que chez les contrôles 50,7% (72/142) avaient une notion de tuberculose dans la famille. Chez les cas, 80.2% (57/71) ne respectaient pas les heures de prise de médicaments tandis que chez les contrôles 43.6% (62/142) ne respectaient pas les heures de prise de médicaments. Dans l'ensemble, 32.4% (69/213) patients avaient interrompu le traitement versus 67.6% (144/213) qui n'avaient pas interrompu le traitement. Chez les cas, 59.1% (42/71) avaient interrompu le traitement tandis que chez les contrôles, 19% (27/142) avaient interrompu le traitement. 59.4% (41/69) avaient comme raison d'interruption du traitement la rupture de stock au niveau de la structure de prise en charge, 17.4% (12/69) avaient d'autres raisons que celles retenues dans cette étude; 13.0% (9/69) étaient des effets secondaires et le manque de nourriture représentaient 10% (7/69) Dans l'ensemble, 34.3% (73/213) avaient fait un échec de traitement et 65.7% n'avaient pas fait d’échecs de traitement. Chez les cas, 76% (54/71) patients avaient un échec de traitement tandis que chez les contrôles 13.3% (19/142) avaient un échec du traitement En ce qui concerne la connaissance de la multi résistance, 9.6%(10/104) connaissaient la définition de la multi résistance et 90.4%(94/104) ne connaissaient pas la définition de la multi résistance. Chez les cas, 8.5% des enquêtés (6/70) étaient diabétiques tandis que chez les contrôles 3.3% étaient diabétiques. Dans cette sous population (les cas), 9.8% des enquêtés (7/71) avaient passé un temps en prison tandis que chez les contrôles 4.2% d'enquêtés avaient fait la prison.

**Tableau 3:** Profil clinique de la population étudiée

Variables	Cas N = 71(%)	Témoins N = 142(%)	Total (%)
**Antécédent TBC**			
Oui	70(99)	130(92)	200(93,9)
Non	1(1)	12(8)	13(6,1)
**Antécédent de tabagisme**			
Oui	12(17)	19(13)	31(14,6)
Non	59(83)	123(87)	182(85,4)
**Antécédent d'alcool**			
Oui	23(32)	47(33)	70(32,9)
Non	48(68)	95(67)	143(67,1)
**Connaissances sur la TBC**			
Oui	64(90)	116(82)	180(84,5)
Non	7(10)	26(18)	33(15,5)
**Attitude de la famille vis-à-vis du malade**			
Avec attention	68(96)	135(95)	203(95,3)
Avec méfiance	3(4)	7(5)	10(4,7)
**Notion de TBC dans la famille**			
Oui	51(72)	72(51)	123(57,7)
Non	20(28)	68(49)	88(41,3)
**Connaissances DOT**			
Oui	51(72)	119(84)	170(79,7)
Non	20(28)	20(16)	39(18,3)
**Pas de respect des heures de prise**			
Oui	57(80)	5(4)	62(29,1)
Non	14(20)	137(96)	151(70,9)
**Connaissances sur le calendrier des examens de contrôle**			
Oui	61(86)	130(92)	191(89,7)
Non	10(14)	12(8)	22(10,3)
**Appréciation de la prise en charge au CSDT**			
Bien	25(35)	32(23)	57(26,8)
Mauvaise	2(3)	0(0)	2(0,9)
Très bien	44(62)	110(77)	154(72,3)
**Interruption de Traitement momentanée**			
Oui	42(59)	27(19)	69(32,4)
Non	29(41)	115(81)	144(67,6)

**Tableau 4:** Profil clinique de la population étudiée (suite)

Variables	Cas N = 71(%)	Témoins N = 142(%)	Total (%)
**Raisons de l'interruption**			
Effets secondaires	4(10)	5(18,5)	9(13,0)
Manque de nourriture	2(5)	5(18,5)	7(10,1)
Rupture au niveau du centre de santé	26(61)	15(56)	41(59,4)
Autre	10(24)	2(7)	12(17,4)
**Echec de traitement**			
Oui	54(76)	19(13)	73(34,3)
Non	17(24)	123(87)	140(65,7)
**Connaissance sur la tuberculose multi résistante**			
Oui	49(69)	58(41)	107(50,2)
Non	22(31)	84(59)	106(49,8)
**Signification de la tuberculose multi résistante pour la malade**			
Résistance à la RIF et ETB	0(0)	2(4)	2(1,9)
Résistance à la RIF et INH	1(2)	9(16)	10(9,6)
Résistance à tous les antituberculeux	16(33)	30(55)	46(44,2)
Ne sait pas	32(65)	14(25)	46(44,2)
**Antécédents ou présence de la tuberculose multi résistantetuberculpulmonaire à Microscopie positive (NC TPM + ) e dans la famille**			
Oui	19(28)	8(6)	27(12,9)
Non	49(72)	134(94)	183(87,1)
**Dépistage du VIH durant les 2 dernières années**			
Oui	69(97)	142(100)	211(99,1)
Non	2(3)	0(0)	2(0,9)
**Connaissance du statut sérologique**			
Oui	69(97)	141(99)	210(98,6)
Non	2(3)	1(1)	3(1,4)
**Etat sérologique**			
Négatif	66(96)	140(99)	206(97,6)
Positif	3(4)	2(1)	5(2,4)
**Diabète**			
Oui	6(9)	1(1)	7(3,3)
Non	64(91)	140(99)	204(96,7)
**Prison**			
Oui	7(10)	2(1)	9(4,2)
Non	64(90)	140(99)	204(95,8)

***L'Association entre certaines caractéristiques sociodémographiques et la tuberculose multi-résistante (Tableau 5):*** Le sexe féminin semblait protégé, mais sans signification statistique avec OR = 0.91; (IC [0.51-1.64]) (p = 0,38). La tranche d’âge de 16 à 36 ans n'avait aucun risque de développer la tuberculose multi résistante (OR = 0.8; IC 0.44-1.45). Les gens qui vivaient dans des ménages composés de plus de 10 personnes (OR = 2,75; IC 1.19-6.34; p = 0,009) avaient environ 3 fois le risque de développer la tuberculose multi résistante que ceux qui vivaient dans les ménages composés de moins de 6 personnes. Et la taille de 7à 10 personnes (OR = 0.88; IC 0,44-1,69; p = 0,35) n'avait pas de risque à développer la tuberculose multi résistante. Les divorcés avaient 7 fois plus le risque de développer la tuberculose multi résistante que les mariés (0R = 6,8; IC 1,59-29,55; p = 0,008), les veufs avaient 3 fois plus le risques de développer la tuberculose multi résistante que les mariés mais sans signification statistique (OR = 2,9; IC 0,38-22,52; p = 0,28); les personnes qui avaient un niveau d’étude primaire avaient 5 fois plus de risque de développer la tuberculose multi résistante que celles du niveau secondaire (OR = 5,1; IC 1,17-22; p = 0,03); les personnes qui avaient un niveau d’étude universitaire couraient 2 fois plus le risque de développer la maladie que ceux qui avaient un niveau d’étude secondaire ( OR = 2,3; IC 1,04-5,4; p = 0,01). Les personnes qui avaient un repas jour avaient 2 fois plus le risques de développer la tuberculose multi résistante que celles qui avaient trois repas par jour mais sans signification statistique (OR = 1,6; IC 0,68-4,02; p = 0,13).

**Tableau 5:** Caractéristiques sociodémographiques et risque de multi résistance

Variables	Cas N=71(%)	Témoin N=142(%)	95%OR	IC	p
**Sexe**					
Féminin	26 (37)	55 (39)	0.9139	0.51-1.64	0,38
Masculin	45 (63)	87 (61)	1		
**Tranche d'âge (ans)**					
16 à 36	43 (61)	93 (65)	0,8	0,44-1,45	0,24
37 à 73	28 (39)	49 (35)	1		
**Profession**					
Chauffeur	5 (0,1)	7 (5)	1,8	0,45-7,23	0,20
Commerçant	3 (4.2)	7 (5)	1,09	0,23-5,19	0,44
Fonction Libérale	8 (11,2)	12 (8)	1,70	0,52-5,54	0,12
Fonctionnaire	9 (13)	23 (16)	1		
Autre	21 (30)	38 (27)	1.41	0,5-4,0	0,47
Sans profession	25 (35.2)	55 (39)	1,16	0,47-2,86	0,38
**Taille Ménage**					
Moins de 6	34 (48)	76 (54)	1		
7 à 10	21 (30)	53 (37)	0,88	0,46-1,69	0,35
Plus de 10	16 (23)	13 (9)	2,75	1,19-6,34	0,009***
**Etat Matrimonial**					
Célibataire	44(62)	82 (58)	1,5	0,81-3,05	0,08*
Divorce	7(10)	3 (2)	6,8	1,59-29,55	0,008***
Marie	17(24)	50 (35)	1		
Union Libre	1(1,4)	5 (4)	0,5	0,06-5,39	0,53
Veuf	2(3)	2(1,4)	2,9	0,38-22,52	0,28
**Niveau d'études**					
Aucun	0 (0)	1 (0,7)	1,91	0,16-22	0,51
Humanitaire	31(43)	51 (36)	2,4	1,12-5,5	0,01**
Primaire	5(7)	4(3)	5,1	1,17-22	0,03
Secondaire	11 (15)	45(32)	1		
Universitaire	24 (34)	41(29)	2,3	1,04-5,4	0,01**
**Source de revenus**					
Oui	26(37)	59(42)	0,81	0,45-1,46	0,24
Non	45(63)	83(58)	1		
**Nombre de repas par jour**					
1	20 (28)	25(18)	1,6	0,68-4,02	0,13
2	38 (54)	90(63)	0,8	0,40-1,88	0,36
3	13 (18)	27(19)	1		

OR=Odd ratio, 95%IC=Intervalle de confiance à 95%, p=valeur-p

**** p < 0.001

*** p < 0.01

** p < 0.05

* p < 0.10

***Association entre profil clinique et la tuberculose multi-résistante (Tableau 6):*** Les personnes qui avaient un antécédent de tuberculose avaient 6 fois plus le risque de développer la tuberculose multi résistante (OR = 6,46; IC 0,82-50,72; p = 0,03); les participants à notre étude qui avaient des antécédents de tabagisme montraient une association entre la tuberculose multi résistante et tabagisme, sans que le tabagisme ne soit un facteur de risque (OR = 1,31; IC 0,59-2,89; p = 0,24). La consommation d'alcool ne constituait pas un facteur de risque de la tuberculose multi résistante (OR = 0,96; IC 0,52-1,77; p = 0,46). Ceux qui vivaient dans les familles où il y avait une notion de la tuberculose avaient 2 fois plus le risques de développer la tuberculose multi résistante mais cette association était sans signification statistique (OR = 2,4; IC 1,3-4,45; p = 0.002). Les tuberculeux qui n'avaient pas respecté les heures de prise durant le traitement de la première ligne étaient 11 fois plus exposés à développer la tuberculose multi résistante (OR = 11,1; IC 38-324; p = 0,00). La connaissance du calendrier des examens de contrôle constituait un facteur protecteur contre la survenue de la tuberculose multi résistante (OR = 0,56; IC 0,23-1,37; p = 0,02). Les patients qui avaient interrompu le traitement étaient 6 fois plus exposés à développer la tuberculose multi résistante. (OR = 6,1; IC 3,27-11,6; p = 0,00). Par ailleurs, notre étude montre que les patients qui avaient présenté un échec de traitement aux médicaments de première ligne avaient 20 fois plus le risque de développer la tuberculose multi résistante (OR = 20,5; IC 9,92-42,6; p = 0,00). Les personnes qui avaient un antécédent de tuberculose multi résistante dans la famille avaient 6,4 fois plus le risque de développer la maladie (OR = 6,4; IC 2,67-15,79; p = 0,00). Les diabétiques avaient 13 fois plus le risque de développer la maladie (OR = 13,1; IC 1,54-111,2; p = 0,005). Les personnes qui avaient passé un séjour en prison avaient 7,6 fois le risque de développer la tuberculose multi résistante (OR = 7,6; IC 1,5-37,88; p = 0,007).

**Tableau 6:** Profil clinique et risque à la tuberculose multi-résistante

Variables	Cas N = 71(%)	Témoins N = 142(%)	OR	95%IC	p
**Antécédent TBC**	70(99)	130(92)	6,46	0,82-50,72	0,03**
**Antécédent de tabagisme**	12(17)	19(13)	1,31	0,59-2,89	0,24
**Antécédent d'alcool**	23(32)	47(33)	0,96	0,52-1,77	0,46
**Connaissances sur la TBC**	64(90)	116(82)	2,04	0,84-4,98	0,054*
**Attitude de la famille vis-à-vis du malade**					
Avec attention	68(96)	135(95)	1,17	0,29-4,68	0,55
Avec méfiance	3(4)	7(5)	1	
**Notion TBC dans la famille**	51(72)	72(51)	2,4	1,3-4,45	0,002***
**Connaissance DOT**	51(72)	119(84)	0,45	0,22-0,91	0,015**
**Pas de respect des heures de prise**					
Oui	57(80)	5(4%)	111	38-324	0,00****
Non	14(20)	137(96%)	1		
**Connaissance sur le calendrier des examens de contrôle**					
Oui	61(86)	130(92%)	0,56	0,23-1,37	0,10
Non	10(14)	12(8%)	1		
**Appréciation de la prise en charge**					
Mauvais	2(3)	0(0%)	7,4	0,7-73	0,08*
Bien	25(35)	32(23%)	1,9	1,02-3,6	0,02**
Très bien	44(62)	110(77%)	1		
**Interruption de Traitement moment**					
Oui	42(59)	27(19)	6,1	3,27-11,6	0,00****
Non	29(41)	115(81)	1		
**Echec du traitement**	54(76)	19(13)	20,5	9,92-42,6	0,00****
**Connaissances sur la TBC multi-résistante**					
Oui	49(69)	58(41)	3,2	1,76-5,9	0,00****
Non	22(31)	84(59)			
**Antécédents ou présence de multi résistance dans la famille**					
Oui	19(28)	8(6)	6,4	2,67-15,79	0,00****
Non	49(72)	134(94)	1		
Sérologie Positif	3(4)	2(1)	0,31	0,05-1,92	0,19
Diabète	6(9)	1(1)	13,1	1,54-111,2	0,005***
Prison	7(10)	2(1)	7,6	1,5-37,88	0,007***

OR = Odd ratio, 95%IC = Intervalle de confiance à 95%, p = valeur-p

**** p < 0.001

*** p < 0.01

** p < 0.05

* p < 0.10

***Modélisation de facteurs de risque de la tuberculose multi résistante:*** Les facteurs de risque de la tuberculose multi-résistante sont représentés dans le Tableau 7. Les patients qui avaient respecté les heures de prise des médicaments pendant leur traitement de première ligne semblaient protégés contre la tuberculose multi résistante (OR= 0,01; IC 0,0047-0,0063; p = 0,00). Ceux qui avaient présenté un échec de traitement avaient 5 fois plus le risque de développer la maladie (OR = 5,5; IC 1,73-17,44; p = 0,00). En outre, le fait d'avoir dans la famille des anciens ou nouveaux tuberculeux constituaient un facteur de risque de développer la tuberculose multi résistante (OR= 6,4; IC 2,67-15,79; p = 0,00). Les personnes qui avaient passé un séjour en prison avaient 19 fois plus le risques de contracter la tuberculose multi résistante (OR = 18,8; IC 1,27-277,54; p= 0,032). De même, les malades tuberculeux qui avaient interrompu leur traitement couraient 3 fois plus le risque de développer la tuberculose multi résistante (OR= 2,78; IC 0,88-8,79; p= 0,081).

**Tableau 7:** Modélisation de facteurs de risque de la tuberculose multi résistante.

Variables		Analyse bivariée			Régression logistique (Multi-variée)		
		OR	IC à 95%	p	ORa	IC à 95%	p
Non-respect des heures de prise des médicaments	Non	1					
	Oui	111	38-324	0,00*	0,02	0,00-0,06	0,00****
Echec de traitement (traitement antérieur	Non	1					
	Oui	20,5	9,9-42,6	0,00*	5,50	1,7-17,4	0,00****
Connaissances de la tuberculose multirésistante	Non	1					
	Oui	3,2	1,8-5,9	0,00*	7,56	2,12-26,96	0,00****
Antécédents ou présence de la tuberculose mutirésistante dans la famille	Non	1					
	Oui	6,4	2,7-15,8	0,00*	6,40	2,67-14,79	0,00****
Prison	Non	1					
	Oui	7,6	1,5-37,9	0,00*	18,82	1,27-277,54	0,03**
Interruption de traitement	Non	1					
	Oui	6,1	3,3-11,6	0,00*	2,72	0,88-8,79	0,08*

OR = Odd ratio, ORa= Odd ratio adjusté, 95%IC = Intervalle de confiance à 95%, p = valeur-p

**** p < 0.001

*** p < 0.01

** p < 0.05

* p < 0.10

## Discussion

La tuberculose multi-résistante constitue incontestablement un réel problème de santé publique dans plusieurs régions du monde [7–18]. Dans notre étude, nous avons pu mettre en évidence de nombreux facteurs de risque de la tuberculose multi-résistante à Kinshasa en République Démocratique du Congo.

***Interruption de traitement:*** Dans notre étude, les malades tuberculeux qui interrompent leur traitement sont 3 fois plus exposés à développer une tuberculose multi-résistante que ceux qui adhèrent au traitement sans interruption. Ceci corrobore les observations d'Ahmad et coll. sur les facteurs de risque de la tuberculose multi résistante au Pakistan. En effet, ces derniers ont trouvé que l'interruption du traitement exposait les patients 15 fois plus à la tuberculeuse multi-résistante [19].

***Echec de traitement (traitement antérieur):*** Nous avons trouvé dans cette étude que les patients qui avaient une notion antérieure du traitement avaient 6 fois plus le risque de développer la tuberculeuse multi-résistante (OR = 5,5 IC 1,7-17,4). Ce résultat corrobore avec les résultats des études menées par Ahmad et coll. et Casal et coll. (OR adj.= 4,2; IC 1,1-15,4 et OR =2,6; IC 1,57-4,41) [19, 20]; Skrahina et coll. au Belarus ont montré que l'historique de traitement antérieur pour la tuberculose représentait le principal facteur de risque indépendant pour la tuberculose multi-résistante (OR: 6,1; intervalle de confiance à 95%, IC: 4,8 - 7,7) [21]. Suárez-Garcia et coll. ont montré dans une étude menée à Madrid que le traitement antérieur constitue un facteur de risque de la tuberculose multi-résistante (OR = 3,44; IC 1,58-7,50) [22]. Ces observations qui corroborent les nôtres démontrent à suffire que les malades qui avaient déjà été traités pour tuberculose présentaient un facteur de risque plus élevé de développer une tuberculose multi-résistante que ceux qui n'avaient jamais été traités.

***Connaissances sur la tuberculose multirésistante:*** Marahatta et coll. ont trouvé que les gens qui ne connaissaient pas la tuberculose multi-résistante étaient 10 fois plus exposés à développer la multi résistance que ceux qui avaient des notions sur la multi résistante tuberculeuse (OR= 9,6; IC 3,3-27,8) [18]. Dans notre étude, nous avons trouvé que les patients qui avaient des connaissances sur la tuberculeuse multi-résistante étaient ceux-là même qui étaient en contact avec les malades tuberculeux multi résistants. Ces derniers étaient donc de fait plus exposés.

***Respect des heures de prise de médicaments:*** Les patients qui respectaient les heures de prise des médicaments pendant leur traitement de première ligne semblaient protégés contre la tuberculose multi résistante. Ces observations corroborent celles des autres auteurs [17, 19].

***Notion d'incarcération (la prison):*** Dans la présente étude, le passage en prison constituait un risque de contracter une tuberculose multi résistante. Ceci se justifie par les conditions de vie précaires, la promiscuité et l'insalubrité (contact avec souches déjà résistantes également rapportés par d'autres études [2, 21–26]. Skrahina et coll par exemple montraient que les autres facteurs de risque indépendants comprenaient l′infection par le virus d′immunodéficience humaine (VIH) et l′alcoolisme [21]. Parmi les autres facteurs de risque, Law et coll. Rapportait, contrairement à nos observations, que l’âge (notamment les jeunes) était un facteur de risque majeur, de même que le fait de vivre sous dépendance financière [23]. Une étude menée par Suárez-Garcia et coll. à Madrid, montrait que la tranche d’âge de 45-64 ans était 3 fois plus exposée à la tuberculose multi-résistance, de même que l'abus de l'alcool représentait un facteur de risque majeur [22].

## Conclusion

Notre étude démontre à suffisance que la tuberculose multi-résistante représente encore un problème majeur de santé publique dans certaines régions du monde et plus particulièrement à Kinshasa en République Démocratique du Congo. L’émergence de la tuberculose multi résistante pourrait pourtant être évitée par la mise en place des stratégies de diagnostic et de traitement appropriées [27].

### Etat des connaissance sur le sujet

- La tuberculose représente un problème majeur de santé publique. Chaque année on compte environ 9 millions de nouveaux cas de tuberculose et près de 2 millions de personnes meurent de cette maladie;
- Chaque année, près de 440 000 personnes contractent une tuberculose multi résistante et 150 000 personnes décèdent de cette forme de la maladie.

### Contribution de notre étude a la connaissance

- La tuberculose multi-résistante représente un problème majeur de santé publique à Kinshasa en République Démocratique du Congo;
- L’émergence de la tuberculose multi résistante pourrait pourtant être évitée par la mise en place des stratégies de diagnostic et de traitement appropriées.
